# A control banding method for chemical risk assessment in occupational settings in France

**DOI:** 10.3389/fpubh.2023.1282668

**Published:** 2023-12-13

**Authors:** Abir Aachimi, Florian Marc, Nathalie Bonvallot, Frederic Clerc

**Affiliations:** ^1^Department of Pollutant Metrology, Institut National de Recherche et de Sécurité (INRS), Paris, France; ^2^Department of Expertise and Technical Consulting, Institut National de Recherche et de Sécurité (INRS), Paris, France; ^3^Univ Rennes, EHESP, Inserm, Irset (Institut de recherche en santé, environnement et travail), Rennes, France

**Keywords:** chemical risk assessment, control banding, chemical product, industrial hygiene, priority of intervention

## Abstract

**Background:**

This study describes a method whose aim is to help companies assess the chemical occupational risks related to labeled products and industrial chemical emissions. The method is intended to be used by industrial hygienists at the scale of one company. Both inhalation and cutaneous exposure routes are taken into account.

**Methods:**

The method relies on a control-banding scheme. A work situation is described by exposure parameters such as the process or the local exhaust ventilation and by the hazard of the product. Each possible value of the parameters is associated with a “band,” which is associated with an integer value. The multiplication of these values results in a score, which represents a priority for intervention. The higher the score, the more the situation warrants investigation for implementing prevention measures, such as chemical substitution and the addition of local exhaust ventilation. To simplify communication, the priority is associated with a colored priority band: red for “very high priority,” orange for “high priority,” and green for “moderate priority.” The priority bands are computed for all work situations performed in a company.

**Results:**

An example of the use of this method is described in a French façade insulation company.

**Conclusion:**

A tool named Seirich was developed to implement this method and promote good practices for helping industrial hygienists in the prioritization of interventions for reducing chemical risk in France.

## 1 Introduction

Occupational health and safety consists of identifying, assessing, prioritizing, and reducing health risks related to exposure to workplace hazards to ensure the safety of employees. In the specific case of chemical risk assessment, a four-step approach is commonly used: identification of the hazard, characterization of the hazard, exposure assessment, and risk characterization (United States Environmental Protection Agency).^1^ In this context, the combination of hazard and exposure data available at the workplace is used. The most accurate way to assess risk is, first, to identify all chemical products found at the workplace and estimate their potential adverse effects with dose–response relationships and, second, to measure workers’ personal exposure through biomonitoring or atmospheric sampling according to Landberg et al. (1). Nevertheless, this approach is often difficult to practically implement by companies due to the lack of competencies, information, and resources. Indeed, the time and money required to conduct exposure measurements within the normative constraints (2) and the many uncertainties associated with the characterization of the products’ potential hazards are not always tractable and even suitable for the size of a company using thousands of chemical products. The “control banding” method can be used as an alternative solution as it uses simplified and more accessible parameters.

Control banding is a qualitative method to assess and manage workplace risks. It consists of matching the “class” for health hazards, exposure potential, and risk mitigation measures. The result of this matching is the generation of a “risk band” that represents the level of risk, which helps the hygienist prioritize and determine prevention action plans as described in Zalk and Nelson (3) and Zalk and Heussen (4). According to Naumann et al. (5), this method was first developed in the 1980s within the pharmaceutical industry to ensure the safety of workers regarding the use of products for which little information was available. To make this method user-friendly and accessible to all companies and to determine an appropriate control strategy for occupational risks, several tools were then developed. As an example, 30 years ago, the UK Health and Safety Executive developed “COSHH Essentials” described in Brooke (6) and Garrod et al. (7) and in the Health and Safety Executive (8) guidance, which is a control-banding tool that determines, through advice and guidance, a control approach to monitor substances that may affect workers’ health. More recently, in 2008, in the context of a Dutch program to reinforce the working conditions policy on hazardous substances, the web-based tool “Stoffenmanager,” described by Cherrie et al. (9) and Marquart et al. (10), was developed to identify chemical hazards and control exposure in the workplace. The hazard banding scheme consists of allocating substances to particular hazard groups based on their toxicological classification and labeling under the CLP regulation, as mentioned by Garrod et al. (7). In 2010, “EMKG” (Einfaches Maßnahmenkonzept Gefahrstoffe) was developed by the German Federal Institute for Occupational Safety and Health (11). As with the other tools, EMKG offers a simple approach to evaluate occupational risks and identify management measures requiring only a minimal number of input parameters.

**Figure 1:**
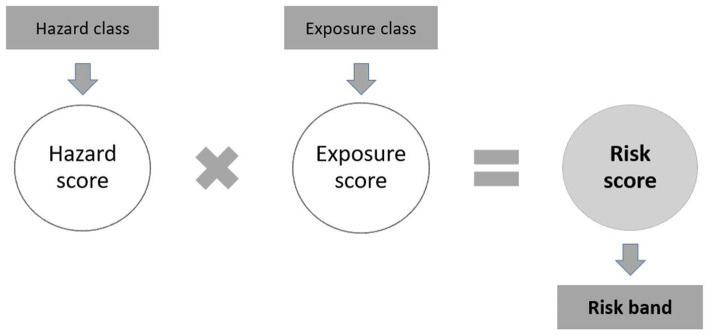
Principles of assessment for chemical risks using the control-banding method.

In 2005, the French National Research and Safety Institute for occupational risk prevention (12), in collaboration with the National Prevention and Protection Centre (CNPP), developed a simplified control banding method described by Vincent et al. (13). The method is intended to be used by anyone with minimal knowledge of chemical risks, using simple and easily accessible parameters. This method evaluates the chemical risks resulting from the potential hazard and exposure to the products used during a task. Later, in 2008, the EU CLP regulation was introduced: the method was updated to support the “H” hazard statements instead of the “R-phrases.” The method is therefore always based on a qualitative assessment of chemical risks, and the output is a relative prioritization of products and industrial chemical emissions for each task performed in the company. The aim of this prioritization is to sort work situations that warrant investigation for implementing prevention measures. Concretely, a hazard band and score are assigned to each product used with regard to the “H” hazard statements. Then, an exposure band and score are assigned, based on sub-scores for each descriptive parameter influencing exposure (process, protective equipment, etc.). Finally, the hazard score and the exposure score are multiplied, and the resulting score is a relative prioritization of the chemical product (Figure 1).

In the first part of this article, the control banding method mostly used by French companies is described. In the second part of this article, a case study of a French insulation and house façade repair company is presented. The workstation chosen for the assessment was the “installation of thermal insulation,” which includes numerous tasks conducted with different products used or emitted.

## 2 Materials and methods

The proposed control banding method has a broad domain of applicability. Since it focuses on the chemical products (a mixture of substances), it allows us to prioritize any CLP-labeled chemical product used in the company, whatever their toxicity since the starting point is the H statements. The chemical products not submitted to the CLP regulations (for example, cosmetics, food products, or waste) and the chemical industrial emissions can also be prioritized. The method consists of three main steps: (I) assignment of the hazard class and score; (II) assignment of the exposure class and score; and (III) calculation of the priority score and assignment of the colored priority band: red for “very high priority,” orange for “high priority,” and green for “moderate priority.” This method has to be followed by the set-up of a prevention action plan to eliminate or reduce the risks threatening the health and safety of employees.

### 2.1 Step 1: assignment of the hazard class and score

In a preliminary task, a map of working areas, workstations, and tasks performed at the company must be prepared. Then, the chemical hazards for each task can be inventoried. For each product, the hazard may be related to a labeled product covered by the European labeling regulation (CLP; i.e., paints, inks, and solvents), a product not covered by the CLP labeling (i.e., flour, sugar, and cosmetic products) or industrial chemical emissions during a particular process without a precise description of products (i.e., wood sanding dust or welding fumes). The hazard is expressed as a hazard class and its corresponding score is expressed as an integer. The hazard class is attributed differently depending on the nature of the chemical:

• For the labeled products covered by CLP labeling, the hazard class is determined through the H and EUH statements available in the SDS or on the product label. Each H or EUH statement is associated with a hazard score according to gravity and potential for immediacy of effect mentioned by the statement. If a product has several hazard statements, the most severe is considered. An overview of the hazard classification for the inhalation route is presented in Table 1. The same principle is used for dermal exposure (data not shown).

**Table 1:** Overview of inhalation hazard classification according to gravity and potential for immediacy of effect.

Inhalation hazard statement according to the CLP regulation	Hazard class
No CLP classification	Very low
Products with moderate local effects, e.g., irritants	Low
Products with acute or chronic moderate toxicity, products with severe local effects, e.g., corrosive, and cutaneous sensitizer products	Medium
Products with immediate effects, products with acute or chronic severe toxicity, e.g., carcinogenic products	High
Products with lethal effects or immediate severe systemic effects, e.g., respiratory sensitizers	Very high

• For the chemical products not covered by CLP labeling and the industrial chemical emissions, the hazard is defined by a consensus of a group of experts in the field of chemical risk prevention. The substances emitted, their toxicity and reactivity, as well as their generation are considered to determine these hazard classes.

In both cases, the assignment of hazard classes process was conducted over months by a group of +20 experts in the field of chemical risk prevention. The results are directly inspired by those from the Health and Safety Executive (HSE), 2008 (8) and in the end very similar to those proposed by (14).

### 2.2 Step 2: assignment of the exposure class and score

**Figure 2:**
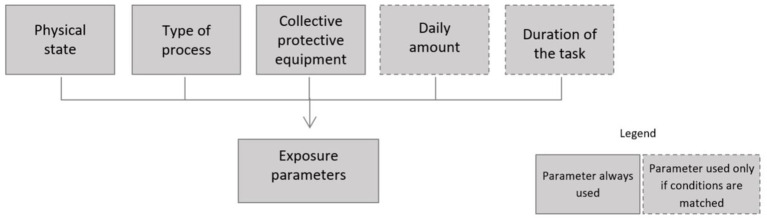
Exposure characterization parameters for the inhalation route.

For the inhalation route, five parameters are needed to evaluate the exposure score (Figure 2). The different modalities of these parameters and their relative classification are listed in Table 2.

**Table 2:** Modalities and classes for the inhalation exposure parameters.

Parameters	Description		Class
Physical state	Solids	Pellets, chips, and solids with little brittleness	Low
		Powder or grains (e.g., crystallized sugar)	Medium
		Fine powder, airborne dust generation during handling (e.g., powdered sugar, flour, and plaster)	High
	Liquids	Vapor pressure lower than 500 Pa	Low
		Vapor pressure between 500 and 10,000 Pa	Medium
		Vapor pressure above 10,000 Pa	High
	Gas	Usually in a pressurized bottle	High
Process	Enclosed	Any process that is completely contained	Very low
	Enclosed but regularly opened	Any process that is confined but can be opened during the filling, emptying, or control phases	Low
	Open	Any process where the material is localized without specific dispersion and without specific containment	Medium
	Dispersive	Any process, which by the energy deployed or the absence of containment generates emissions into the working atmosphere	High
Collective protective equipment	Indoors	Fume cupboard	Very low
		Other local exhaust ventilation (extractor hood, extraction slit, and extraction table)	Low
		General ventilation	Medium
		No extraction device	High
	Outdoors (natural ventilation)		Medium
Daily amount	< 10 g		Very low
	[10–100 g]		Low
	[100–1 kg]		Medium
	[1–10 kg]		High
	≥ 10 kg		Very high
Duration	< 15 min		Very low
	[15 min–1 h]		Low
	[1–4 h]		Medium
	≥ 4 h		High

The modalities are ranked in relative terms, with regard to each parameter, and ranked from “very low” to “high”.

• The physical state can be “liquid,” “solid,” or “gas.” It is used to describe the potential of the substance to become airborne. When it is a liquid, this potential is defined by the vapor pressure, and in this case, the temperature of use and the boiling temperature can be used (EUSES, European Union System for the Evaluation of Substances, available^2^). When it is a solid, including powders, the potential is related to the dustiness: the finer the powder, the higher the potential. When it is a gas, the potential is always at the maximum level because gases are considered to generate maximum exposure.

• The type of process is used to define the level of dispersion of the product in the workplace. It can be defined by using the REACH process reference framework (PROC) defined in the European Chemicals Agency (15) guidance or by using the four modalities defined in the Technical Guidance Document on Risk Assessment (16).

• Collective protective equipment concerns the installation of ventilation controls and local exhaust ventilation, which contributes to the protection of employees’ health. These measures help to reduce the levels of exposure to chemicals for employees.

• The daily amount corresponds to the amount of product used during a specific task over a day (8 h) or during a work sequence. The daily amount is only used with dispersive processes; it defines the amount of product dispersed voluntarily in the work atmosphere.

• The duration of the task performed by the employee is considered when the most severe hazard occurs after repeated exposure over time (chronic exposure, i.e., carcinogenic products). On the contrary, the duration of exposure is not considered when the most severe hazard occurs after acute exposure (i.e., highly toxic products that can cause immediate irreversible effects).

**Figure 3:**
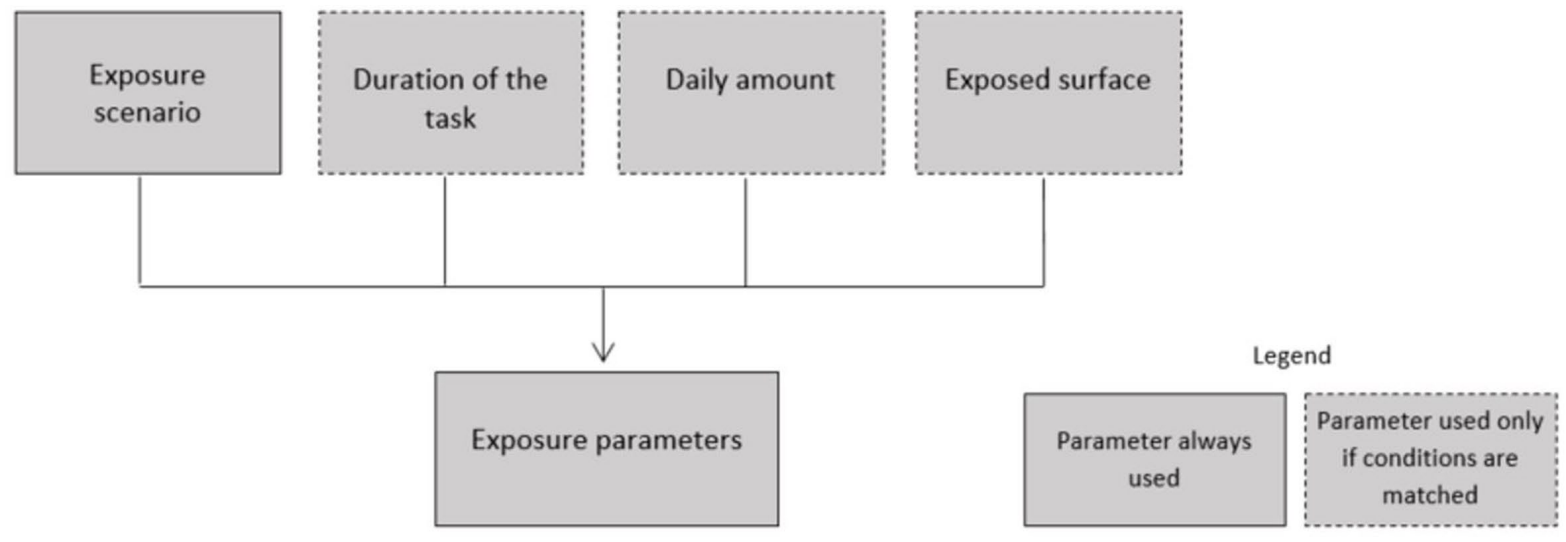
Exposure characterization parameters for dermal exposure.

For the dermal route, which includes both the skin and eyes, four parameters are needed to assess the exposure (Figure 3). The different modalities of these parameters and their relative classification are listed in Table 3.

**Table 3:** Modalities and classes for dermal exposure parameters.

Parameters	Description	Class
Exposure scenario	No possible contact of the product with the body	Very low
	Possible contact of the product with a part of the body (e.g., handling of a cloth soaked with a product or tools contaminated by a product)	Low
	Possible generation of splashes or aerosols (e.g., projection of drops during spill operations and projection of oil mists by rotating machines)	Medium
	Possible immersion of a part of the body in the product (e.g., manual placing or removal of parts in chemical baths, during degreasing, and rinsing operations)	High
Exposed surface	One hand	Very low
	Both hands	Low
	Lower or upper limbs	Medium
	The whole body or face	High
Daily amount	< 10 g	Very low
	[10–100 g]	Low
	[100 g–1 kg]	Medium
	[1 kg–10 kg]	High
	≥10 kg	Very high
Duration	<15 min	Very low
	[15 min–1 h]	Low
	[1–4 h]	Medium
	≥ 4 h	High

The modalities are ranked in relative terms, with regards to each parameter, and ranked (from “very low” to “high”).

• The exposure scenario corresponds to the nature of the operations performed by the employee. There are four modalities for the exposure scenario describing a part of the exposure level.

• The exposed surface corresponds to the total surface area of skin that can be exposed to the product without considering personal protective equipment.

• The daily amount is taken into account in the same way as for the inhalation route. This parameter is considered when the effects appear because of exposure through skin penetration (systemic effects). It is not used when the product produces local effects.

• The duration is considered in the same way, with the same modalities, as for the inhalation route.

An integer value is allocated to each of the abovementioned entry parameter modalities, and the exposure score is the multiplication of these integer values.

### 2.3 Step 3: calculation of the priority score and assignment of the priority band

The priority score is calculated by multiplying the hazard score and the exposure score: one for the inhalation route and another for the dermal route. The value attributed to the hazard score has the most important weight compared to the exposure score. Then, the inhalation route priority band and the dermal route priority band are assigned with regard to their respective priority score: “moderate priority” (green color), “high priority” (orange color), and “very high priority” (red color). The priority bands are calculated for each work situation in the company. Then, the work situations are sorted according to their respective priority.

## 3 Results. Example of application for a workplace: installation of thermal insulation

In 2019, a visit to a company specialized in the insulation and repair of house façades was conducted. The company was identified following a request made by a hygienist from the French public health insurance service, explaining that the director of this company wanted to evaluate and establish an action plan to reduce the potential chemical risks within his company. The aim of the visit was to contact the director, understand his needs, and explain the usefulness of the method and its usage. To do this and to facilitate the task, the authors suggested carrying out an assessment of one of the company’s workstations, from the inventory to the action plan, according to the three steps defined above.

### 3.1 Step 1: assignment of the hazard class and score

Different workstations using chemical products were identified in the company: scaffolding, installation of thermal insulation, repair and renovation of façades, painting, and coating. The workstation chosen for the assessment was the “installation of thermal insulation” due to the numerous tasks conducted with different products used or emitted. Information concerning the tasks and the products was collected during the company visit. Table 3 represents the eight tasks performed with the inventory of labeled products and industrial chemical emissions.

### 3.2 Step 2: assignment of exposure class and score

The details regarding the calculation of priority level via both inhalation and dermal routes are shown in Table 4 for all labeled products used in the workstation. For industrial chemical emissions, the determination details are shown in Table 5.

**Table 4:** Tasks performed in the workstation with labeled products and industrial chemical emissions.

Task	Labeled product	Chemical emissions
Surface preparation and installation of the starting rails (sanding and drilling)	–	Dust emissions
Hot wire cutting of polystyrene insulation boards	–	Plastic combustion fumes
Treatment of protruding angles (reinforcing strips)	–	Dust emissions
Filling fractional gaps	Expanding foam and silicone sealant	–
Adding wefts	Epoxy bonding mortar	–
Adding fixative between lattice and plaster	Primer	–
Façade coating	Surface hardener, bonding resin, porosity regulator, and façade coat	–
Finishes and removal of residues	Hydrochloric acid; 2 waterproofing products, façade coat	–

**Table 5:** Hazard and exposure data and levels assigned by the method to calculate the inhalation and dermal chemical priority scores for all labeled products used in the workstation.

	Inhalation route				Dermal route			
	**Task: Filling fractional gaps** The residual fractional gaps in the polystyrene boards are filled with chemical products, depending on the size of the gap. This operation is done manually by the worker, either with an aerosol of expanding foam or with a silicone gun.							
	**P1: Silicone sealant**		**P2: Expanding foam**		**P1: Silicone sealant**		**P2: Expanding foam**	
	*Data*	*Level*	*Data*	*Level*	*Data*	*Level*	*Data*	*Level*
***Hazard***	No CLP statement	Very low	*- May cause respiratory irritation* *- May cause allergy or asthmatic symptoms or breathing difficulties if inhaled* *- Suspected of causing cancer* *- May cause harm to breast-fed children* *- May cause damage to organs through prolonged or repeated exposure*	Very high	No CLP statement	Very low	*- Causes skin irritation* *- May cause an allergic skin reaction* *- Causes serious eye irritation*	Medium
***Exposure***	Physical state: Paste, considered in liquid category	Low	Physical state: Foam, considered in the liquid category	Low	Exposure scenario: Possible contact of the product with a part of the body	Low	Exposure scenario: Possible contact of the product with a part of the body	Low
	Process: dispersive	High	Process: dispersive	High	Exposed surface: both hands	Low	Exposed surface: both hands	Low
	CPE: Outdoor work	Medium	CPE: outdoor work	Medium	Duration: Not required	–	Duration: Not required	–
	Duration: Not required	–	Duration: Not required	–	Daily amount: 3 L	High	Daily amount: 7 L	High
	Daily amount: 3 L	High	Daily amount: 7 L	High				
***Priority***	**Moderate**		**Very high**		**Moderate**		**Very high**	
	**Task: Adding lattice** All polystyrene boards on the whole façade are covered by a metallic lattice, which is sealed on the façade with mortar. This lattice will support the coating. This operation is done manually by the worker. He applies the mortar from a mason’s through with a trowel. Then the lattice is sealed into the mortar.							
	**P: Epoxy bonding mortar**				**P: Epoxy bonding mortar**			
	*Data*		*Level*		*Data*		*Level*	
***Hazard***	No hazard statement		Very low		- *Causes skin irritation* *- Causes serious eye damage*		Medium	
***Exposure***	Physical state: Fine powder		High		Exposure scenario: Possible contact of the product with a part of the body		Low	
	Process: Open		High		Exposed surface: Both hands		Low	
	CPE: Outdoor work		Medium		Duration: Not required		–	
	Duration: Not required		–		Daily amount: 279 kg		Very high	
	Daily amount: 279 kg		Very high					
***Priority***	**High**				**High**			
	**Task: Adding fixative between lattice and plaster** Once the lattice is installed and the mortar is dry, a layer of primer is applied manually with a roller. The primer is used from a bucket.							
	**P: Primer**				**P: Primer**			
	*Data*		*Level*		*Data*		*Level*	
***Hazard***	-*May produce an allergic reaction*		High		*-May produce an allergic reaction*		High	
***Exposure***	Physical state: viscous liquid, considered in liquid category		Low		Exposure scenario: Possible contact of the product with a part of the body		Low	
	Process: Open		High		Exposed surface: Both hands		Low	
	CPE: Outdoor work		Medium		Duration: Not required		–	
	Duration: Not required		–		Daily amount: 8 kg		High	
	Daily amount: 8 kg		High					
***Priority***	**Very high**				**High**			
	**Task: Façade coating** First, the worker prepares the mixture. The different products are poured manually into a bucket and mixed with a paint mixer. The mixture is then manually applied with a trowel and roller.							
	**P1: Surface hardener**		**P2: bonding resin**		**P1: Surface hardener**		**P2: bonding resin**	
	*Data*	*Level*	*Data*	*Level*	*Data*	*Level*	*Data*	*Level*
***Hazard***	*-May produce an allergic reaction*	High	May produce an allergic reaction.	High	-*May produce an allergic reaction*	High	-May produce an allergic reaction	High
***Exposure***	Physical state: liquid	Low	Physical state: liquid	Low	Exposure scenario: Possible contact of the product with a part of the body	High	Exposure scenario: Possible contact of the product with a part of the body	High
	Process: dispersive	High	Process: Dispersive	High	Exposed surface: both hands	High	Exposed surface: both hands	High
	CPE: outdoor work	Medium	CPE: outdoor work	Medium	Duration: not required	–	Duration: not require	–
	Duration: not required	–	Duration: not required	–	Daily amount: 18 kg	Very high	Daily amount: 14 kg	Very high
	Daily amount: 18 kg	Very high	Daily amount: 14 kg	Very high				
***Priority***	**High**		**Very high**		**High**		**High**	
	**P3: Porosity regulator**		**P4: Façade coat**		**P3: Porosity regulator**		**P4: Façade coat**	
	*Data*	*Level*	*Data*	*Level*	*Data*	*Level*	*Data*	*Level*
***Hazard***	*-May cause respiratory irritation.*	Low	*-May produce an allergic reaction.*	High	*-Causes skin irritation.* *Causes serious eye damage.*	Medium	*-May produce an allergic reaction.*	High
***Exposure***	Physical state: fine powder	High	Physical state: paste, considered in the liquid category.	Low	Exposure scenario: Possible contact of the product with a part of the body	Low	Exposure scenario: Possible contact of the product with a part of the body	Low
	Process: dispersive	High	Process: dispersive	High	Exposed surface: the whole body or face	High	Exposed surface: the whole body or face	High
	CPE: outdoor work	Medium	CPE: outdoor work	Medium	Duration: not required	–	Duration: not required	–
	Duration: not required	–	Duration: not required	–	Daily amount: 5 kg	High	Daily amount: 270 kg	Very high
	Daily amount: 5 kg	High	Daily amount:270 kg	Very high				
***Priority***	**High**		**High**		**High**		**Very high**	
	**Task 8: Finishes and removal of residues** The façade is manually ground and sandpapered where needed. The waterproofing product is applied manually with a roller, and hydrochloric acid is used to remove the residues. More façade coat is applied manually with a smaller trowel where needed to obtain a smooth finish.							
	**P1: Hydrochloric acid**		**P2: Waterproofing product (1)**		**P1: Hydrochloric acid**		**P2: Waterproofing product (1)**	
	*Data*	*Level*	*Data*	*Level*	*Data*	*Level*	*Data*	*Level*
***Hazard***	*-May cause respiratory irritation*	Low	No CLP statement	Very low	*-Causes severe skin burns and eye damage*	High	*-Causes skin irritation* *-May cause an allergic skin reaction* *-Causes serious eye damage*	Medium
***Exposure***	Physical state: liquid	Low	Physical state: fine powder	High	Exposure scenario: Possible contact of the product with a part of the body	Low	Exposure scenario: possible contact of the product with a part of the body	Low
	Process: dispersive	High	Process: dispersive	High	Exposed surface: the whole body or face	High	Exposed surface: the whole body or face	High
	CPE: outdoor work	Medium	CPE: outdoor work	Medium	Duration: not required	–	Duration: not required	–
	Duration: not required	–	Duration: not required	–	Daily amount: 3 L	High	Daily amount: 18 kg	Very high
	Daily amount: 3 L	High	Daily amount: 18 kg	Very high				
***Priority***	**High**		**High**		**Very high**		**High**	
	**P3: Waterproofing product (2)**		**P4: Façade coat**		**P3: Waterproofing product (2)**		**P4: Façade coat**	
	*Data*	*Level*	*Data*	*Level*	*Data*	*Level*	*Data*	*Level*
***Hazard***	No CLP statement	Very low	*-May produce an allergic reaction.*	High	*- Causes skin irritation* *- May cause an allergic skin reaction* *- Causes serious eye damage*	Medium	*-May produce an allergic reaction.*	High
***Exposure***	Physical state: fine powder	High	Physical state: paste, considered in the liquid category.	Low	Exposure scenario: possible contact of the product with a part of the body	Low	Exposure scenario: possible contact of the product with a part of the body	Low
	Process: dispersive	High	Process: dispersive	High	Exposed surface: the whole body or face	High	Exposed surface: the whole body or face	High
	CPE: outdoor work	Medium	CPE: outdoor work	Medium	Duration: not required	–	Duration: not required	–
	Duration: not required	–	Duration: not required	–	Daily amount: 18 kg	Very high	Daily amount: 270 kg	Very high
	Daily amount: 18 kg	Very high	Daily amount:270 kg	Very high				
***Priority***	**High**		**High**		**High**		**Very high**	

*CPE corresponds to collective protective equipment.

### 3.3 Step 3: calculation of the priority score and assignment of the priority band

**Figure 4:**
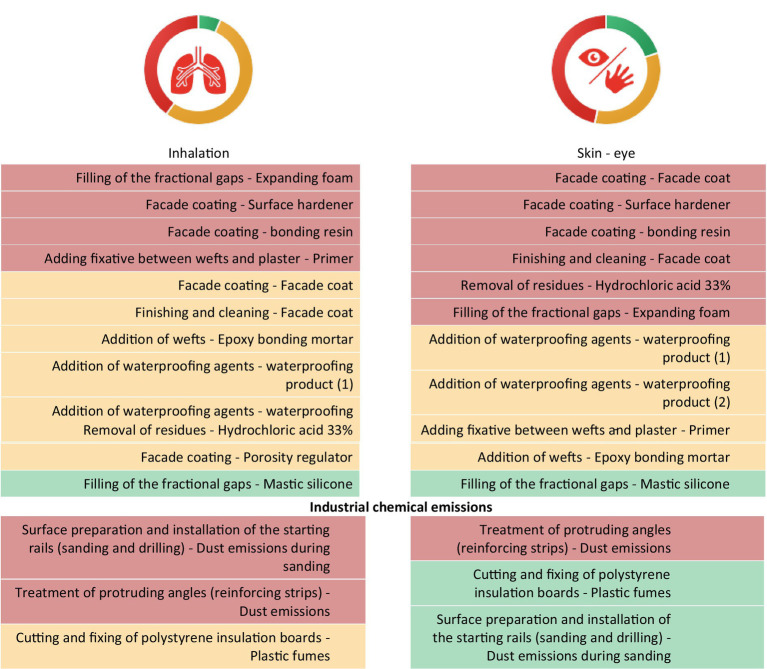
Prioritization results according to the risk scores for inhalation and dermal (skin and eyes) exposure for labeled products and industrial chemical emissions.

Figure 4 represents the sorted list of products used during each task according to their respective inhalation and dermal priority bands.

Regarding the priorities for the inhalation route illustrated in this example, the four products used during tasks with “very high inhalation priority” were as follows: (1) the expanding foam used to fill fractional gaps, (2) the surface hardener, (3) the bonding resin used for the façade coating, (4) and the primer used as a fixative between the lattice and the plaster. Moreover, two industrial chemical emissions also showed “very high inhalation priority”: the dust emitted (1) during the surface preparation and installation of the starting rails and (2) during the treatment of protruding angles. The next seven products and the plastic combustion fumes released during the cutting of polystyrene insulation boards had a high priority, as shown in orange in Figure 4. Regarding priorities related to the dermal route, six products were used during tasks with “very high priority” as follows: (1&4) the façade coat, (2) the surface hardener, (3) the bonding resin, all used for the façade coating and the finishing; (5) the hydrochloric acid used for the finishing task; and (6) the expanding foam used to fill the fractional gaps. Moreover, one industrial chemical emission also showed “very high dermal priority”: the dust released during the treatment of protruding angles.

The aim of this prioritization at a company is to guide the development and the follow-up of a preventive or corrective action plan helpful to reduce occupational risks for the most problematic situations. Therefore, to help the company determine the appropriate actions, an occupational hygienist from the French public health insurance service was asked to review the results. A precise action plan was established. In particular, the substitution of the expanding foam, the surface hardener, and the bonding resin were required because their use was considered a “very high priority” for inhalation and dermal routes. The dust emitted during the surface preparation and the treatment of protruding angles presented a very high inhalation priority. Since these tasks are performed outdoors, the use of collective protective equipment is not applicable. For this reason, the use of personal respiratory protective equipment is highly recommended to avoid the risks related to this task. In addition, given the very high priority via the dermal route for the treatment of protruding angles, the use of dermal protective equipment (goggles and gloves) is recommended during the treatment of protruding angles (Table 6).

**Table 6:** Hazard and exposure data and levels assigned by the method to calculate the inhalation and dermal chemical risk scores for all industrial emissions released in the workstation.

	Inhalation route		Dermal route	
	**Task: Surface preparation and installation of the starting rails (sanding and drilling)** The façade is ground where needed, metallic fasteners are installed in drilled holes, and metallic rails are installed horizontally and vertically on the façade. Different handheld tools can be used (driller, grinder, or perforator) and also manual hammer and chisel. The fasteners and rails are installed manually			
	**Data**	**Level**	**Data**	**Level**
***Hazard***	Dust emissions	High	Dust emissions	Very low
***Exposure***	Physical state: not required for emissions	–	Exposure scenario: possible generation of splashes or aerosols (e.g., projection of drops during spill operations and projection of oil mists by rotating machines).	Medium
	Process: dispersive	High	Exposed surface: the whole body or face	High
	CPE: outdoor work	Medium	Duration: 1 h–4 h	Medium
	Duration: 1 h–4 h	Medium	Daily amount: not required for emissions	–
	Daily amount: not required for emissions	–		
***Priority***	**Very high**		**Moderate**	
	**Task: Hot wire cutting of polystyrene insulation boards** The polystyrene boards are installed on the rails, and some of them need to be cut to the correct size on the ground. This operation is done with a special hot wire tool.			
	**Data**	**Level**	**Data**	**Level**
***Hazard***	Plastic combustion fumes	High	Plastic combustion fumes	Very low
***Exposure***	Physical state: not required for emissions	–	Exposure scenario: possible generation of splashes or aerosols (e.g., projection of drops during spill operations and projection of oil mists by rotating machines).	Medium
	Process: dispersive	High	Exposed surface: the whole body or face	High
	CPE: Outdoor work	Medium	Duration: 15 min–1 h	Low
	Duration: 15 min–1 h	Low	Daily amount: not required for emissions	–
	Daily amount: not required for emissions	–		
***Priority***	**High**		**Moderate**	
	**Task: Treatment of protruding angles (reinforcing strips)** Once the polystyrene boards and coat are applied, some strips have to be installed on the angles so there is no fragmentation of edges. The strips are cut manually, the edges are ground and sandpapered where needed.			
	**Data**	**Level**	**Data**	**Level**
***Hazard***	Dust emissions	High	Dust emissions	High
***Exposure***	Physical state: not required for emissions	–	Exposure scenario: Possible generation of splashes or aerosols (e.g., projection of drops during spill operations and projection of oil mists by rotating machines).	Medium
	Process: dispersive	High	Exposed surface: The whole body or face	High
	CPE: outdoor work	Medium	Duration: 1 h–4 h	Medium
	Duration: 1 h–4 h	Medium	Daily amount: not required for emissions	–
	Daily amount: not required for emissions	–		
***Priority***	**Very high**		**Very high**	

*CPE corresponds to collective protective equipment.

## 4 Discussion

The method can be used to attribute an intervention priority to work situations involving exposure to chemical products through inhalation and dermal routes. This method’s domain of applicability extends to almost all types of products except for non-specific powders (i.e., without CLP statements). Moreover, the priorities can be attributed according to any type of working situation, whatever the task or the process involved.

To evaluate the hazard, the labeled products are associated with hazard classes based on their H and EUH statements. In addition to the major sources mentioned previously in this article, other tools such as Stoffenmanager, EMKG, and Ecetoc TRA described in Bögi et al. (17) use similar schemes. As there is no reference methodology for assigning each hazard statement to a specific band, the assignments made by each tool are different with the use of different rules. In these tools, the carcinogenic, mutagenic and reprotoxic hazards are often associated with the most severe hazard band. In the proposed methodology, the most severe band refers to lethal acute toxicity. A similarity between these tools is the classification of products capable of causing harm to unborn babies or impacting negatively on fertility, which is classified just after the classification of the most severe hazards. The qualitative identification of hazards includes subjectivity related to the use of expert judgments that are based on training and experience. As for the risks, hazard perceptions depend on many variables, such as personal and socio-demographic aspects, and the professional experience of the evaluators, as noted by Skjong et al. (18). Since different institutions and individuals develop these different tools, this may explain the differences in the hazard ranking tables. Moreover, as control banding is a relative method, the prioritization of the hazard into five classes helps to rank and prioritize products according to their level of dangerousness, but the least severe class in the hazard table does not mean that the hazard represented is not considerable.

To assess the exposure, most models cited above evaluate the concentration of substances contained in the products in the worker’s breathing zone. This concentration is compared to occupational exposure limits (OELs) to assess the chemical risk, expressed as “above OEL” or “below OEL.” By comparison, in this method, a risk assessment is conducted regarding the use of products and not only the substances. This is considered more convenient to field practitioners since workers are usually exposed to a mixture of substances that constitute the products and not to the substances individually. However, even if this method provides a risk assessment of the products used in the company, it does not replace the regulations related to the monitoring of occupational exposure, which, in all cases, require employers to carry out exposure measurements for regulated substances that are considered to be of concern and to compare them with occupational exposure limit values.

In this method, the input parameters must be easily accessible. The parameters that are difficult to access, but which are essential for evaluation, are simplified. For example, the air change rate is represented by the type of mitigation system used and the product volatility, which is defined by the vapor pressure, and can be estimated by using the boiling point and the temperature of use if the vapor pressure is not available. Moreover, the frequency of use of products is not considered relevant because the aim is to evaluate the risk resulting from the exposure of the worker during the task (at the time he/she performs the work operation) and not at the workplace in general. The number of exposed workers in the workplace is an important parameter in risk management. However, regardless of the number of workers in the area of potential damage, the severity of this damage must be the same: this parameter does not influence the risk assessment. The volume and/or the surface area of the work zone is also not considered because it is not easily accessible to all users.

Even relying on a robust control banding methodology, chemical risk assessment remains difficult. Some specific issues related to particular substances can be improved. First, when the product evaluated does not have an SDS or is not classified according to the CLP regulation for health hazards, the chemical risk given by the method is always at the minimum level. Among these unclassified products, there are powder products with non-specific effects (i.e., calcium carbonate, amorphous silica, and alumina). This type of chemical agent can cause various respiratory system pathologies resulting from pulmonary overload or carcinogenic, allergenic, or irritant substances, as mentioned in a report by the French Agency for Food, Environmental and Occupational Health and Safety – ANSES (19). The method underestimates these effects since the products do not have a classification according to the CLP regulation. This methodology limitation was reported during its use and a solution is currently being developed to rectify it. Second, endocrine disruptors are difficult to identify and the evaluation of their effects on health is a scientific challenge and an important public health issue as noted by ANSES (20) and the ECHA (21). Despite these uncertainties, a preventive approach should be implemented to limit the workers’ exposure to the lowest possible level, particularly pregnant women or women of childbearing age, as recognized in the INRS (22) report. This issue and a solution to address it will be proposed in the future. Third, the quality of the assessment depends on the quality of the information from the SDSs. Meanwhile, SDSs often do not provide complete or accurate information. For example, the physicochemical properties (vapor pressure) are sometimes missing. More importantly, the product’s descriptions of health effects need more improvement within the European Chemicals Agency (23) report. This lack of data in the SDSs mainly concerns powders, especially nanometric ones. These powders are not always well identified in the SDSs and information on their composition or their potential hazards is often not available. This leads to a misjudged risk assessment for this type of product. Hodson et al. (24) evaluated the reliability and accuracy of a sample of SDS specific to engineered nanomaterials. Their evaluation showed that their information quality is not sufficient to provide adequate data on the inherent health and safety hazards of engineered nanomaterials. Thus, the use of SDSs alone to characterize the products’ hazards could be considered as a limitation because even though each user is asked to verify the adequacy and SDS updates, the method is not able to confirm their accuracy and the quality of data provided on the product’s effects.

This method is implemented in a software named “Seirich,” which was developed by the INRS in partnership with the French Ministry of Labor, national health insurance, and French professional organizations. In addition to the control banding chemical risk assessment, Seirich software guides users in the development and follow-up of a preventive or corrective action plan to reduce risks at work. A risk assessment is provided for fire and explosion hazards. The software also offers regulation information and good practices to guide the user in the implementation of preventive actions. It is available free of charge on the web^3^ (French and English languages).

## 5 Conclusion

For more than 20 years, and particularly since the coming into force of the EU CLP regulation in 2015 (for mixtures), a constant evolution of the presented method has been conducted, with several improved versions implemented in the Seirich software. This involves either considering regulatory updates, introducing ergonomic evolutions, or adding new features. Currently, this method is widely used for occupational chemical risk assessment in France with more than 30,000 users. The INRS is committed to promoting this tool and ensuring its continuous improvement. This tool represents a very important step in the risk prevention process by allowing the identification and evaluation of chemical risks to which employees are exposed in the workplace. This must be followed by the implementation of a specific prevention action plan based on the results obtained, with the aim of eliminating or reducing the identified risks as much as possible. Finally, to allow foreign companies to use it easily, this tool is also available in an English version but is still adapted to French regulations.

## Data availability statement

Publicly available datasets were analyzed in this study. This data can be found at: https://doi.org/10.1080/15459624.2021.2023161.

## Author contributions

AA: Data curation, Investigation, Methodology, Software, Validation, Visualization, Writing – original draft. FM: Conceptualization, Formal analysis, Funding acquisition, Methodology, Project administration, Resources, Validation, Writing – review & editing. NB: Conceptualization, Project administration, Resources, Supervision, Validation, Writing – review & editing. FC: Conceptualization, Formal analysis, Funding acquisition, Project administration, Resources, Supervision, Validation, Writing – review & editing.

## Footnotes

^1^https://www.epa.gov/risk/human-health-risk-assessment

^2^https://echa.europa.eu/fr/support/dossier-submission-tools/euses

^3^https://www.seirich.fr/
